# Additive Manufacturing of Caffeic Acid-Inspired Mineral Trioxide Aggregate/Poly-ε-Caprolactone Scaffold for Regulating Vascular Induction and Osteogenic Regeneration of Dental Pulp Stem Cells

**DOI:** 10.3390/cells10112911

**Published:** 2021-10-27

**Authors:** Ni Tien, Jian-Jr Lee, Alvin Kai-Xing Lee, Yen-Hong Lin, Jian-Xun Chen, Ting-You Kuo, Ming-You Shie

**Affiliations:** 1 Department of Medical Laboratory Science and Biotechnology, China Medical University, Taichung 406040, Taiwan; t6719@mail.cmuh.org.tw; 2 Department of Laboratory Medicine, China Medical University Hospital, Taichung 404332, Taiwan; 3 School of Medicine, China Medical University, Taichung 406040, Taiwan; D33977@mail.cmuh.org.tw (J.-J.L.); Leekaixingalvin@gmail.com (A.K.-X.L.); d21996cmuh@gmail.com (J.-X.C.); 4 Department of Plastic & Reconstruction Surgery, China Medical University Hospital, Taichung 404332, Taiwan; 5 x-Dimension Center for Medical Research and Translation, China Medical University Hospital, Taichung 404332, Taiwan; roger.lin0204@gmail.com; 6 The Ph.D. Program for Medical Engineering and Rehabilitation Science, China Medical University, Taichung 406040, Taiwan; 7 Department of Surgery, China Medical University Hospital, Taichung 404332, Taiwan; 8 The Master Program for Biomedical Engineering, China Medical University, Taichung 406040, Taiwan; bill78moa456@gmail.com; 9 School of Dentistry, China Medical University, Taichung 406040, Taiwan; 10 Department of Bioinformatics and Medical Engineering, Asia University, Taichung 41354, Taiwan

**Keywords:** angiogenesis, caffeic acid, human dental pulp stem cell, osteogenesis, surface modification

## Abstract

Mineral trioxide aggregate (MTA) is a common biomaterial used in endodontics regeneration due to its antibacterial properties, good biocompatibility and high bioactivity. Surface modification technology allows us to endow biomaterials with the necessary biological targets for activation of specific downstream functions such as promoting angiogenesis and osteogenesis. In this study, we used caffeic acid (CA)-coated MTA/polycaprolactone (PCL) composites and fabricated 3D scaffolds to evaluate the influence on the physicochemical and biological aspects of CA-coated MTA scaffolds. As seen from the results, modification of CA does not change the original structural characteristics of MTA, thus allowing us to retain the properties of MTA. CA-coated MTA scaffolds were shown to have 25% to 55% higher results than bare scaffold. In addition, CA-coated MTA scaffolds were able to significantly adsorb more vascular endothelial growth factors (*p* < 0.05) secreted from human dental pulp stem cells (hDPSCs). More importantly, CA-coated MTA scaffolds not only promoted the adhesion and proliferation behaviors of hDPSCs, but also enhanced angiogenesis and osteogenesis. Finally, CA-coated MTA scaffolds led to enhanced subsequent in vivo bone regeneration of the femur of rabbits, which was confirmed using micro-computed tomography and histological staining. Taken together, CA can be used as a potently functional bioactive coating for various scaffolds in bone tissue engineering and other biomedical applications in the future.

## 1. Introduction

Mineral trioxide aggregate (MTA) was first introduced in the 1990s as ProRoot MTA and soon became a revolutionary material in endodontics [1]. The composition of MTA is a calcium silicate-based bioactive ceramics, mainly composed of dicalcium silicate, tricalcium silicate, bismuth oxide, and silicon dioxide [2]. MTA had since been extensively researched upon and is used in numerous applications such as dentin repairs and regeneration, apexification, pulp capping and pulpotomies [3,4,5]. ProRoot MTA was a mixture of bismuth oxide and cement, where the cement has an important role to play in ensuring success of the MTA. The cement polymerizes in a moist environment, thus ensuring tight fit of gap and stability of material [6]. In addition, MTA was reported to have antimicrobial characteristics, and high biocompatibility and bioactivity due to its capability to deposit hydroxyapatite layers on its surface [1]. The hydroxyapatite layer is an important interface between the MTA and the tooth which enhances biomineralization and tissue regeneration. MTA has also been confirmed by numerous studies in its capability to stimulate proliferation and differentiation of dental stem cells [7]. Furthermore, MTA contains calcium oxide which converts to calcium hydroxide when in contact with humidity, thus creating a strong alkaline microbacterial environment ideal for tissue regeneration [8].

Surface modification of scaffolds can endow biomaterials with the necessary biological targets for activation of specific downstream functions [9]. Various types of surface modification approaches have been explored, including plasma treatment, surface oxidation and photo-induced grafting [10]. A previous study conducted by Tu et al. showed that MTA could be coated with caffeic acid (CA), a natural polyphenol, via simple immersion techniques. This surface modification concept was derived upon observation of the attachment capabilities of mussels on walls of docks. Numerous natural antioxidants such as caffeic acid have since been used for modification of synthetic biomaterial in order to enhance bioactivity of scaffolds [11]. Caffeic acid (CA), also known as 3-(3,4-dihydroxyphenyl)-2-propenoic acid, is a commonly used polyphenol due to its strong antioxidative, antibacterial and anti-inflammatory properties [12]. The study conducted by Tu et al. showed that CAMTA cements had enhanced, mechanical, odontogenic, angiogenic and immunosuppressive characteristics as compared to pure MTA [13]. In their study, CAMTA had 1.6× higher mechanical properties and led to a decrease in IL-1β and IL-1RA levels at all time points. Angiogenesis-related factors, Von Willebrand factor (vWF), vascular endothelial growth factors (VEGF), and odontogenic-related proteins were significantly enhanced with the CA modification, thus strongly indicating the possibility of surface modification for dentin regeneration. In addition, Chiang et al. coated CA onto chitosan scaffolds and showed that such a modification improved mechanical properties, biodegradability and cytocompatibility of chitosan scaffolds [14]. Furthermore, the modified chitosan scaffolds had higher antibacterial effects against Staphylococcus aereus than neat chitosan scaffolds.

An important aspect of tissue regeneration is angiogenesis, which is the formation and branching of new blood vessels from existing vessels [15]. There are two main types of angiogenesis driven by the same signaling molecules; regenerative vessels, which are functional, and interconnected vessels supplying regenerating tissues and tumor vessels, which are made up of irregular and immature vessels [16,17]. VEGF is often the first consideration to promote vascularization and angiogenesis inside the scaffold in the tissue engineering environment [18]. In the environment, VEGF is the most potent growth factor on angiogenesis and is thus commonly used in tissue engineering for promoting angiogenesis. In addition, Kuttuppan et al. combined VEGF, fetal growth factors and bone-morphogenetic protein 2 (BMP-2), and the initial results similarly showed enhanced angiogenesis with immature vascular networks [19]. Previously, Chen et al. fabricated VEGF-hydroxyapatite scaffolds and it was shown that the presence of VEGF significantly increased vWF and angiopoietin-1 proteins, which led to enhanced angiogenesis and subsequent osteogenesis [20]. However, it was noted that the scaffolds released VEGF in a burst manner instead of having a sustained release. A VEGF-loaded scaffold should have sustained release to sustain angiogenesis and regeneration. Godoy-Gallardo et al. showed that composite scaffolds could be fabricated using polycaprolactone (PCL) and hydroxyapatite with simultaneous multi-layered coating with polydopamine, BMP-2 [21]. In their study, a synergistic relationship between the angiogenic and osteogenic cells were observed and thus proved the importance of a co-culture ecosystem in tissue regeneration.

In this study, we modified MTA with CA, applied the extracts of polycaprolactone (PCL) into CA-modified MTA and fabricated 3D porous scaffolds. First, we considered the physical and chemical properties of the scaffold and speculated that the MTA scaffolds modified by CA would improve its properties. The bioactivity and biocompatibility of CAMTA scaffolds were systematically elucidated by analyzing the adhesion of human dental pulp stem cells (hDPSCs), and subsequently its levels of growth and differentiation. In addition, the CAMTA scaffold was not only shown to rapidly adsorb the VEGF secreted by hDPSCs, but it was also proven to promote cell angiogenesis and osteogenesis. The potential of this design strategy allows the identification of further areas for hard tissue regeneration. Finally, the in vivo femur bone defect regeneration experiments were presented to examine the osteogenic capability of CAMTA scaffolds, and we showed that the scaffolds can significantly improve bone repair at the defect site.

## 2. Materials and Methods

### 2.1. MTA Modification and 3D Scaffold Fabrication

Mineral Trioxide Aggregate (ProRoot MTA; Dentsply, Tulsa, OK, USA) is a common biomaterial used in clinical applications such as dentin regeneration. To modify surface of MTA powder, 10 g of MTA was added to 100 mL of tris(2,2’-bipyridyl) dichlororuthenium(II) hexahydrate (tris-buffer, Sigma-Aldrich, St Louis, MO, USA) to obtain a MTA solution of pH 8.5 and 5 mM concentration. Then, caffeic acid (Sigma-Aldrich, St. Louis, MO, USA) with a concentration of 0, 10, 20 mg/mL was added to the tris-buffer, and the mixture was vigorously stirred (400 rpm) overnight at room temperature for 24 h. Then, the filtrate was collected using 5 µm filter paper and suction filtration and washed with absolute alcohol thrice. Subsequently, the filtrate was dried at 100 °C for 24 h, and grinded using a ceramic grinder to obtain the different concentrations of CA-modification MTA and stored in a dehumidifier cabinet until further usage. Next, a thermoplastic polymer, polycaprolactone (PCL, molecular weight = 43,000–50,000; Polysciences, Warrington, PA, USA) was added to improve the printing quality of CAMTA. The preparation of the composite PCL/CAMTA was briefly described as follows. Firstly, CAMTA powder was ultrasonically dissolved in anhydrous alcohol (0.1 g/mL) for 5 min, followed by 30 min of stirring using a magnetic stirrer. At the same time, the PCL was preheated to 150 °C to obtain a transparent fluid. The CAMTA–alcohol solution was then pipetted dropwise into the PCL solution and stirred until all the alcohol had evaporated. The mixture was then placed into a 100 °C oven overnight to obtain a printable PCL/CAMTA composite. The scaffold was fabricated using GeSiM (BioScaffolder 3.1, GeSiM, Grosserkmannsdorf, Germany). Firstly, the PCL/CAMTA composite was loaded into the dedicated cartridge for GeSiM, and heated to 70–90 °C. The printing parameters were as follows: printing rate was 1 mm/s, printing pressure was 400 kPa, layer height was 0.3 mm, strut length was 0.5 mm and distance between each strut was 0.5 mm. The parameters were presented as a range in this study as each composite requires slight fine-tuning to ensure smooth printing. The scaffolds were first designed using a dedicated software and uploaded into GeSiM as .stl files. Each layer was stacked 90° to one another and stacked upwards to obtain rectangular scaffolds. The different concentrations were recorded as CA0, CA10, and CA20 according to the concentrations of CA-coated MTA.

### 2.2. Physicochemical Properties of the CA-Coated MTA Scaffolds

An EZ Test machine (Shimadzu, Kyoto, Japan) was used to evaluate for the mechanical properties of the scaffolds. The various scaffolds were stretched at a rate of 1 mm/min until the scaffolds fractured. The stress–strain curves were then recorded to evaluate for the maximal compressive properties of the scaffolds. Six scaffolds from each group were evaluated with the average recorded. In addition, Fourier-transform infrared spectrometer at the spectral range of 500 cm^−1^ to 4000 cm^−1^ (FTIR, Vertex 80v, Bruker, Karlsruhe, Germany) was used to evaluate for the functional bonds in each scaffold.

### 2.3. In Vitro Soaking and Bioactivity Assay

The scaffolds were immersed in 37 °C simulated body fluid (SBF) solution. The SBF solution was similar to human blood plasma and consisted of 7.9949 g of NaCl, 0.2235 g of KCl, 0.147 g of K_2_HPO_4_, 0.3528 g of NaHCO_3_, 0.071 of g Na_2_SO_4_, 0.2775 g of CaCl_2_ and 0.305 g of MgCl_2_·6H_2_O in 1000 mL of distilled H_2_O with the pH adjusted to 7.4 using hydrochloric acid and trishydroxymethyl aminomethane. At various time points, the scaffolds were removed from the SBF and characterized the phase composition of CA-coated scaffolds using the X-ray diffractometer analysis (XRD, Shimadzu Corporation, Kyoto, Japan) at the following condition: 1° divergence and scatter slits, receiving slot of 0.10 mm at 20° to 50° and at θ to 2θ with a scan speed of 1°/min. A field emission scanning electron microscope (SEM, JEOL, Tokyo, Japan) was used to observe for the microstructure morphology of the scaffolds.

### 2.4. Cell Proliferation and Morphology

For subsequent studies, the scaffolds were first immersed in 75% ethanol for 20 min to disinfect the scaffolds. Human dental pulp stem cells (hDPSCs) used in this study were from Lonza (PT-5025, Lonza, Basel, Switzerland) and cultured with a commercially available human dental pulp stem cell bullet kit (PT-3005, Lonza, Basel, Switzerland) to passage 3–6 in a 37 °C humidified atmosphere with 5% CO_2_. The hDPSCs were detached using TrypLE™ (Invitrogen, Carlsbad, CA, USA), collected using a hemocytometer, and seeded on various CA-coated MTA scaffolds at a density of 5 × 10^4^ cells/mL in 48-well plate with 1 mL medium per well. After being cultured for different time periods, PrestoBlue assay (Invitrogen, Carlsbad, CA, USA) was used to evaluate for the proliferation of hDPSCs. In brief, after 1, 3, and 7 days of culture, the medium was mixed with PrestoBlue reagent at a ratio of 9:1 for 90 min in a 37 °C incubator. Then, 100 μL of the solution was transferred to new 96-well. hDPSCs cultured on cell plates were used as the control group (Ctl). The optical density of the solution was evaluated at a wavelength of 570 nm using a spectrophotometer (Infinite Pro M200, Tecan, Männedorf, Switzerland).

For cell morphology, the scaffolds were first rinsed with cold PBS and fixed in 4% paraformaldehyde (Sigma-Aldrich, St. Louis, MO, USA) for 30 min. After this, the cells were lysed with 0.1% Triton X-100 (Sigma-Aldrich, St. Louis, MO, USA) in PBS for 15 min. Fluorescent staining was performed by incubating the specimens with phalloidin conjugated to Alexa Fluor 488 (1:500, Invitrogen, Carlsbad, CA, USA) for 2 h in the dark. Then, the specimens were gently rinsed with cold PBS solution to remove excess solution, and DAPI fluorescent dye (Invitrogen, Carlsbad, CA, USA) was used and left to react for 20 min in the dark. The scaffolds were then rinsed with PBS and morphology was observed using a confocal microscope (Leica TCS SP8, Wetzlar, Germany).

### 2.5. VEGF Secretion and Adsorption

The enzyme-linked immunosorbent assay kit (ELISA, KHG0111, Invitrogen, Carlsbad, CA, USA) was used to analyze the level of VEGF secretion at the various time points according to manufacturer’s instructions with a standard curve. In addition, ELISA was used to detect for the amount of VEGF adsorption on the surfaces of the various scaffolds [22]. hDPSCs were detached using TrypLE™, and the scaffolds were then rinsed thrice using PBS containing 0.1% Tween 20. After which, 5% bovine serum albumin (Gibco, Grand Island, NY, USA) in PBS-T was used to conduct cell block for 1 h. The scaffolds were then incubated with 1:500 anti-human VEGF antibody (Abclonal, Woburn, MA, USA) for 3 h at room temperature, rinsed thrice with PBS-T for 5 min and incubated with horseradish peroxidase-conjugated secondary antibodies for 1 h at room temperature whilst shaking. The scaffolds were then rinsed thrice with PBS-T for 10 min, and One-Step Ultra TMB substrate (Invitrogen, Carlsbad, CA, USA) was added to the wells and left to react for 30 min at room temperature in the dark. An equal amount of 2 M H_2_SO_4_ was added to stop and stabilize the oxidation reaction. The colored products were then transferred to new 96-well plates and evaluated using a Tecan Infinite 200^®^ PRO microplate reader at 450 nm (reference 620 nm). The above tests were conducted thrice and the average was recorded. Scaffolds with cells incubated with β-actin antibodies were used as controls.

### 2.6. Angiogenesis and Osteogenesis Assay

The hDPSCs were cultured using angiogenesis/osteogenesis assay kits (StemPro™ angiogenesis differentiation kit/ osteogenesis starter kit; Invitrogen, Carlsbad, CA, USA) for 3, 7 and 14 days for evaluation of angiogenic and osteogenic capabilities. Cells were first lysed using NP40 cell lysis solution (Sigma-Aldrich, St. Louis, MO, USA) and centrifuged at 6000 rpm for 15 min. The secretion of VEGF (KHG0111, Invitrogen, Carlsbad, CA, USA), Angiopoietin-1 (Ang-1, ab99972, Abcam, Cambridge, MA, USA), bone sialoprotein (BSP, MBS261861, MyBioSource, San Diego, CA, USA) and osteocalcin (OC, ab195214, Abcam, Cambridge, MA, USA) from hDPSCs were determined using ELISA with a standard curve. The above tests were conducted thrice and the average was recorded. In addition, the activity of alkaline phosphatase (ALP) activity was evaluated by pNPP (Sigma-Aldrich, St. Louis, MO, USA), according to the manufacturer’s instructions. After reacting with pNPP for 40 min, 5 M NaOH was immediately added to terminate the reaction. Absorbance was examined at 405 nm using a spectrophotometer. Finally, ALP activity was standardized with protein content using the Pierce BCA Protein Assay Kit (BCA, Thermo Fisher Scientific, Waltham, MA, USA).

### 2.7. Rabbit Model of Femoral Bone Defects

All in vivo experimental protocol was approved by the Animal Experimental Ethics Committee of China Medical University in Taichung, Taiwan (CMUIACUC-2019-091). Healthy three months old New Zealand rabbits weighing about 1.8–2 kg were selected for our study. The rabbits were divided into two groups of three rabbits each and one group was implanted with CA20 scaffold, whilst the other group was implanted with CA0 scaffold. The surgical procedures were described in brief. Firstly, chlorhexidine was injected into the rabbits and they were anaesthetized using 100% oxygen and 5% isoflurane. Their hind legs were shaved and disinfected using alcohol and iodine. The incision was made and fascia removed, while avoiding injury to the nerves and blood vessels. Then, a dental drill was used to create a bone defect to the femur of the rabbits. The various scaffolds were then implanted into the defect and sutured layer-by-layer. The rabbits were then monitored closely and given free access to food and water.

### 2.8. Micro-Computed Tomography (µCT) and Histological Analysis

The µCT images were performed to estimate new bone regeneration surround the scaffolds at the defect site. The constructs were analyzed using multi-scale X-ray nano-CT System (SkyScan 2211, Bruker, Belgium) at a voltage of 100 kVp, current of 330 μA, and an output of 20 Watts. Three-dimensional images were reconstructed using an Insta Recon software (Insta Recon, Bruker, Belgium) and analyzed using a software AvizoFire 8.0 (Visualization Sciences Group, Bordeaux, France) to determine the bone volume (BV/TV) and the trabecular thickness (Tb.Th) of the bone. After the µCT scaning analyzed was completed, the entire embedded sample, which included the defect site with the implanted treated CA-coated MTA scaffold and the surrounding native bone tissue, was cut into 6 μm sections using a microtome. The sections were stained with hematoxylin and eosin (H&E, ScyTek Lab, West Logan, UT, USA), Masson’s trichrome stain kit (MT, ScyTek Lab, West Logan, UT, USA), and von Kossa kit (VK, ScyTek Lab, West Logan, UT, USA). The trichrome staining in blue was measured to identify collagen distribution. The Von Kossa was dark brown which means, the mineralization of osteoid tissue and peri-calcified bone was observed. All sample staining was detected by the microscope (BX53, Olympus, Tokyo, Japan).

### 2.9. Data Analysis

A one-way variance statistical analysis was used to evaluate the significance of the differences between the means in the measured data. A Scheffe’s multiple comparison test was used to determine the significance of the deviations in the data for each specimen. In all cases, the results were considered statistically significant if the *p* value < 0.05.

## 3. Results and Discussion

### 3.1. Physical Properties

**Figure 1:**
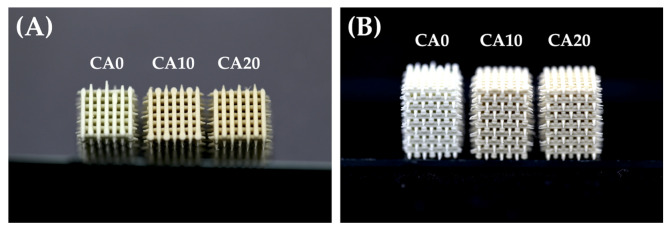
(**A**) The top view and (**B**) side view photographs of various CA-coated MTA scaffolds.

In this study, MTA powders were modified with various concentrations of CA and 3D printed via extrusion printing to fabricate scaffolds that were shown in Figure 1. In fact, there are numerous methods for fabricating a scaffold for tissue engineering such as electrospinning, extrusion printing or stereolithography [23]. In our case, we decided on extrusion printing over the rest of the methods, as it is more suitable for fabricating scaffolds with sufficient mechanical properties and porosity for hard tissue engineering [24,25]. As seen from Figure 1A, the addition of CA resulted in a brownish appearance, whereas neat commercial MTA (CA0) were lighter in color. In addition, all pores and struts were well interconnected and uniform in size (Figure 1B). Sufficient porosity is needed to accommodate cell proliferation and differentiation, which will eventually enhance tissue formation [26]. It is also desirable for a scaffold to have high interconnectivity between pores for uniform cell seeding and distribution, nutrition support and efficient waste removal [27]. A good scaffold for hard tissue engineering should have approximately 100–400 µm of well-interconnected pores to support good tissue regeneration and the addition of CA into MTA did not affect such desired characteristics of the scaffolds. The 3D microenvironment was able to adopt proper structures and cellular interactions, and allow nutrient perfusion to all levels and thus is commonly used for modeling mechanisms of normal physiological processes, tumor biology, tissue regeneration and monitoring of treatment efficacy [28,29]. This initial screen showed that the design of our scaffolds was able to withstand mechanical loading and forces typically experienced during implantation and at the scaffold–tissue interface. One of the major challenges of a good scaffold is achieving initial sufficient mechanical strength so as to support tissue regeneration [30].

**Figure 2:**
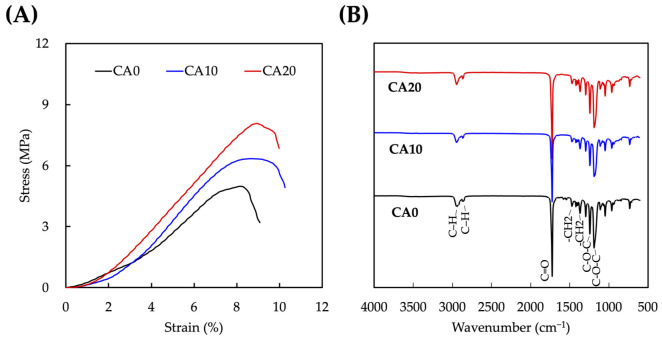
(**A**) Stress–strain curve and (**B**) Fourier transform infrared spectroscopy results of CA0, CA10, and CA20.

The mechanical properties of the various scaffolds were evaluated according to internationally recognized standards and results were as shown in Figure 2A. As mentioned, a good scaffold should have sufficient mechanical strength to withstand surgical implantation procedures and native forces [31]. The results were then recorded and plotted into stress–strain curves. As seen, CA0 had a tensile strength of approximately 4.5 ± 0.3 MP whilst after CA grafting, CA10 and CA20 had 1.25- and 1.55-times higher tensile strength than CA0, respectively. All scaffolds were noted to have steep stress–strain slope, thus indicating that the scaffolds were able to withstand sudden increase in tensile forces before breaking. The tensile strength of CA10 and CA20 were reported to be 5.8 ± 0.4 and 8.1 ± 0.5 MPa, respectively, thus making it an even more ideal candidate for hard tissue engineering [32]. Unalan et al. were the first to report regarding the idea of grafting benzene ring moiety onto scaffolds to improve mechanical strength of scaffolds [33]. In our case, CA is known to contain benzene ring structures, thus further contributing to the increase in mechanical strength of the scaffolds directly proportional to the concentrations of CA coating. FTIR was used to confirm the successful corporation of CA on MTA powders and results were as shown in Figure 2B. As seen, the characteristic peaks of MTA were observed in all groups. Strong bands in the 2800 to 3000 cm^−1^ regions correspond to the C-H- stretching and 1650 cm^−1^ regions to the C=O region. In addition, characteristic asymmetrical stretching peaks were noted at the 1200 to 1400 cm^−1^ region which represents -C12 and C-O-C bonds present in MTA. Interestingly, the peaks of all the scaffolds were noted to be similar even after CA modification. This suggests that CA and MTA were bounded with hydrogen bonds and thus do not reflect any structural changes to the FTIR results. Kaczmarek-Szczepanska et al. fabricated chitosan scaffolds coated with CA and their FTIR results had similar trends to our study [34]. It was hypothesized from the above results that CA-coated MTA substrates could allow us to retain the original benefits of MTA, yet with the additional advantages that CA could bring about for hard tissue regeneration.

### 3.2. Immersion Behaviors

**Figure 3:**
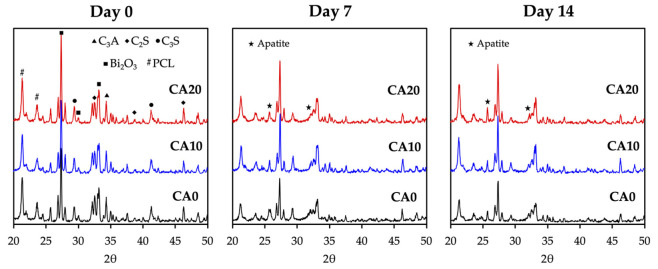
X-ray diffraction (XRD) analysis of CA0, CA10, and CA20 scaffolds before and after the immersion process for 7 and 14 days.

The XRD patterns of the scaffolds on day 0, 7 and 14 of immersion in SBF were as shown in Figure 3. It can be observed that the peaks present in D0 for all groups correspond to the compositions of MTA. C3A, C2S, and C3S correspond to tricalcium aluminate, dicalcium silicate and tricalcium silicate, respectively, and are all critical components of MTA. After immersion for 7 and 14 days, the intensity of MTA-related peaks was decreased, whereas the intensity of apatite were noted at 2θ = 25.9° and 32.5° for all groups [35]. CA20 was noted to have a higher intensity of apatite peaks at both day 7 and 14 of immersion than CS0 and CS10. MTA has been widely explored and studied for the regeneration of apical hard tissues and is currently considered as a standard treatment guideline for apical root lesion after root canal therapy. In general, MTA is considered to be biocompatible and is non-cytotoxic to cells. MTA scaffolds were noted to release Ca, Si and hydroxyl ions, which were all key components in promoting odontogenesis [2]. These released ions interact with P ions in the simulated body fluid to form a layer of hydroxyapatite on the surfaces of scaffolds, which then in turn enhances tooth-material sealing and regeneration. In this study, we attempted to regulate behaviors by modifying MTA with CA to regulate odontogenesis [36]. CA has been widely used in multiple settings due to its strong antibacterial and antioxidative capabilities. Shiu et al. grafted CA onto chitosan scaffolds and showed that the antibacterial and antioxidative capabilities against Staphylococcus Aureus were significantly enhanced [37]. In addition, CA-chitosan scaffolds had a two-fold increase in mechanical properties and strong antitumor capabilities against osteosarcoma cell lines. Together with our XRD results, it was hypothesized that our CA-modified MTA scaffolds had the potential to upregulate osteogenesis, unlike neat MTA scaffolds.

**Figure 4:**
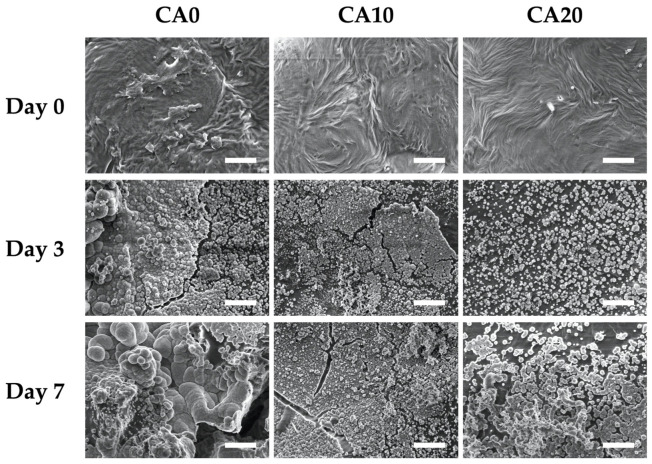
Surface microstructure of the CA0, CA10, and CA20 scaffolds before and after immersion in SBF for 3 and 7 days. The scale bar is 2 μm.

The morphology of the surface precipitates was observed using SEM after day 0, 3 and 7 of immersion and they were as shown in Figure 4. The SEM images of the scaffolds on day 0 showed that all the scaffolds had rough irregular surfaces, with CA20 having more irregular contours than the rest of the groups. Liu et al. reported that material surface roughness has an important role to play in modulating cellular activities [38]. In their study, the bone marrow stem cells were noted to have higher levels of osteogenic capabilities when cultured on micro-rough surfaces than cells cultured on smooth surfaces. Different cell lines were found to have varying preferences for the levels of surface roughness, but in general, odontogenic and osteogenic-related cells were found to have improved regenerative capabilities when cultured on macro-rough surfaces. The SEM images on day 3 and 7 of immersion showed hydroxyapatite precipitation on the surfaces of the various scaffolds. However, CA0 had fewer sparse clusters of nanoparticles, whilst CA10 and CA20 had denser aggregates of hydroxyapatite formation covering a wider surface area than CA0. The resultant hydroxyapatite formation not only enhances biocompatibility and enhances tooth-material sealing, it also has an important role to play in enhancing cellular adhesion and attachment, thus leading to increased cellular activities such as proliferation and differentiation [39]. Therefore, several scientists had attempted to coat or induce hydroxyapatite onto surfaces of biomaterials in order to improve the regeneration capabilities of scaffolds. Therefore, the levels of hydroxyapatite formation on surfaces of ceramic-based scaffolds has been widely accepted to be a predictor marker for subsequent tooth or bone regeneration [40].

### 3.3. Biocompatibility

**Figure 5:**
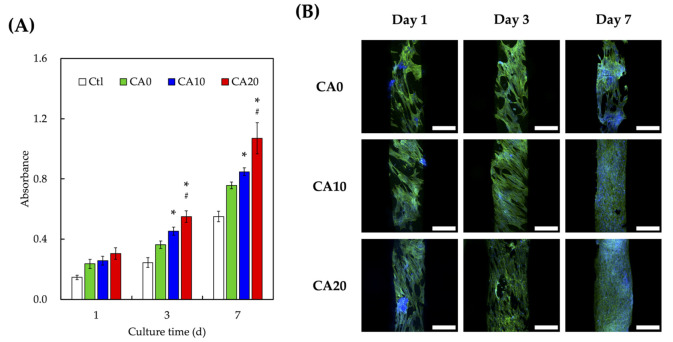
(**A**) Proliferation rate and (**B**) F-actin (green) and nuclei (blue) staining of hDPSCs cultured on different CA-coated MTA scaffolds for 1, 3, and 7 days. * indicates a significant difference (*p* < 0.05) from CA0. # indicates a significant difference (*p* < 0.05) from CA10. Data presented as mean ± SEM; *n* = 6 for each group. The scale bar is 200 μm.

The proliferation rates of hDPSCs were quantified using PrestoBlue and are as shown in Figure 5A. It could be seen that all groups had similar proliferation and viability after 1 day of culture (*p* > 0.05). However, significant differences in proliferation (*p* < 0.05) were noted on CA10 and CA20 scaffolds when compared to CA0 from day 3 onwards. In addition, on both day 3 and 7 of culture, CA20 shown had significantly higher levels of proliferation than CA10 (*p* < 0.05). These results clearly indicated the coating of CA was able to enhance proliferation rates and viability of hDPSCs in a dose-dependent manner [13]. MTA were originally known to release Si ion which act on the Wnt pathway to regulate cellular regeneration and behaviors [7]. With the modification of CA, it could be seen that existing materials in the market could be easily modified in order to potentially enhance its tissue regenerative capabilities. Confocal microscopy for F-actin staining was carried out as shown in Figure 5B. As seen, CA20 had the greatest number of cells at all time points as compared to the other groups. In addition, the F-actin of hDPSCs in CA20 were flatter and well-spread as compared to those in CA0. The F-actin of CA0 were shorter and clustered, thus indicating that the cells were not well-adhered and attached to the surfaces of scaffolds. There were reports stating that the initial quality of cell adhesion could be used as a predictor for the subsequent proliferation and differentiation of cells [41]. Therefore, initial results had shown that the addition of CA made the micro-environment more suitable for cellular adhesion and that CA were non-toxic to cells.

**Figure 6:**
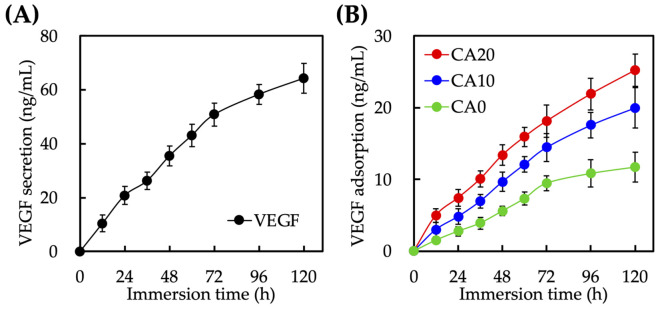
(**A**) VEGF secretion from hDPSCs and (**B**) adsorption on the CA0, CA10, and CA20 scaffold surface for 120 h (*n* = 6).

The levels of VEGF secretion from hDPSCs and levels of VEGF adsorption on the surfaces of scaffolds were as shown in Figure 6. As seen from Figure 6A, there was a steady increase in VEGF in the surrounding fluid during the first 120 h of immersion. There was 20 ng/mL of VEGF after 24 h of culture, which increased three-fold to 60 ng/mL after 120 h of culture. As mentioned, VEGF is an important factor involved in the extracellular matrix remodeling during angiogenesis [42]. In order to enhance angiogenic capabilities of hDPSCs for dentin regeneration, Zhu et al. attempted to transfect VEGF genes into hDPSCs so as to induce higher levels of VEGF secretion to induce subsequent proliferation and angiogenic capabilities [43]. Subsequent results showed that such a method is feasible in in vitro studies to promote angiogenesis. However, our team felt that gene therapy is still in its initial stages and would require more extensive studies for future clinical applications. Therefore, we attempted to modify MTA with CA in order to enhance VEGF adsorption via the influence of external factors, such as surface roughness and involvement of other ionic elements. The amount of VEGF adsorption onto surfaces of scaffolds were also evaluated as shown in Figure 6B. As expected, CA20 had higher levels of VEGF absorption at all time points as compared to CA10 and CA0 (*p* < 0.05). After 24 h of culture, there was 2.8 ± 0.6, 4.8 ± 1.1, and 7.4 ± 1.1 ng/mL of VEGF adsorption on CA0, CA10 and CA20, respectively. In other words, CA20 showed 2.5- and 1.4-times higher levels of VEGF absorption than CA0 and CA10, respectively. It was hypothesized that the addition of CA caused surfaces of scaffolds to become more negatively charged, thus allowing for the positively charged amino acids of VEGF to be adsorbed onto the surfaces of scaffolds. Singh et al. published an article in *Biomaterials* stating that the higher the levels of VEGF adsorption on surfaces of scaffolds, the higher the angiogenic responses of scaffolds [44].

### 3.4. Angiogenesis

**Figure 7:**
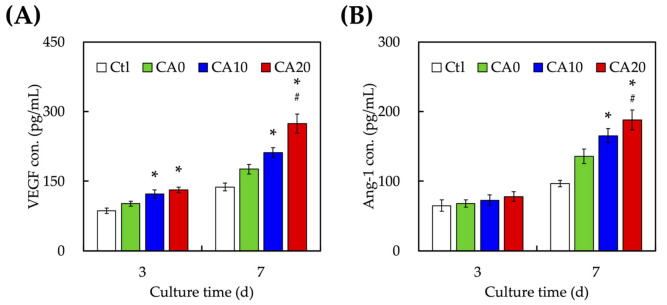
Angiogenesis-related differentiation markers of (**A**) VEGF and (**B**) Ang-1 expression of hDPSCs cultured on CA0, CA10, and CA20 scaffolds for different time points. * indicates a significant difference (*p* < 0.05) from CA0. # indicates a significant difference (*p* < 0.05) from CA10. Data presented as mean ± SEM; *n* = 6 for each group.

Figure 7 shows the cumulative amount of VEGF and Ang-1 released from CA0, CA10 and CA20 after 3 and 7 days of culture to evaluate for the angiogenic capabilities of our CAMTA scaffolds. After 3 days of culture, the amount of VEGF expressed from CA0, CA10 and CA20 was 101.4 ± 5.5, 122.4 ± 8.6 and 131.3 ± 6.1 pg/mL, respectively. CA10 and CA20 were noted to have significantly higher levels of VEGF than CA0 after 3 days of culture. After being cultured for 7 days, the levels of VEGF from CA20 were found to be 1.7- and 1.4-times higher than CA0 and CA10, respectively. Recently, there were numerous studies attempting to load different types of growth factors into scaffolds in order to enhance tissue regeneration [45,46,47]. Li et al. showed that the VEGF-loaded hydroxyapatite-collagen scaffolds significantly enhanced levels of angiogenesis and subsequently osteogenesis in an ischemic limb rat model [48]. Tissue regeneration requires sufficient angiogenesis in order to provide nutrients and for waste removal. VEGF is generally produced during hypoxia and binds to specific receptors on the walls of endothelial cells [49]. By doing so, the vessels undergo characteristic morphological changes, including enlargement of the diameter, basement membrane degradation, thinning of endothelial cell lining, upregulating endothelial number, followed by decreasing the number of pericytes and detachment of pericytes. New blood vessels then sprout from the existing vessels with the assistance of matrix metalloproteinases. On the other hand, Ang-1 acts as a complement to VEGF by mediating interactions between endothelial cells and surrounding mesenchyme. It is also involved in the later stages of vessel maturation and remodeling. Therefore, there were no significant differences in the levels of Ang-1 for all groups on day 3 of culture. However, after 7 days of culture, the levels of Ang-1 for CA0, CA10 and CA20 were 135.5 ± 10.4, 165.1 ± 10.2 and 187.7 ± 14.3 pg/mL, respectively. Numerous studies have proven that Ang-1 expression at the later stages of development was able to increase bone regeneration via increasing expression of osteogenic markers such as bone morphogenetic protein 2 and Runx2 genes. Kakio et al. showed that CA significantly induced VEGF expression by activating HIF-1α in the primary cells [50]. In addition, the presence of CA enhances NF-kβ activation, thus leading to increased VEGF receptors and VEGF secretion of cells. Taken together, our results demonstrated that the CA modifications were able to enhance expressions of VEGF and Ang-1 and allowed for increased adhesions of VEGF and Ang-1 onto surfaces of the scaffolds, thus having the potential to lead to enhanced odontogenesis. Therefore, further tests were required to determine the osteogenic capabilities of CA-modified MTA scaffolds.

### 3.5. Osteogenesis

**Figure 8:**
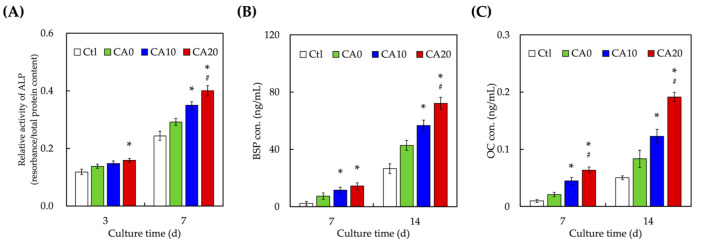
Osteogenesis-related differentiation markers of (**A**) ALP activity (**B**) BSP, and (**C**) OC expression of hDPSCs cultured on CA0, CA10, and CA20 scaffolds for different time points. * indicates a significant difference (*p* < 0.05) from CA0. # indicates a significant difference (*p* < 0.05) from CA10. Data presented as mean ± SEM; *n* = 6 for each group.

The levels of osteogenic-related proteins such as ALP (Figure 8A), BSP (Figure 8B) and OC (Figure 8C) were evaluated by ELISA. CA20 were noted to have significantly higher levels of ALP, BSP and OC from day 3 onwards than CA0. Levels of ALP were 1.6-times higher in the CA20 group on day 7 than in the CA0 group. In addition, CA20 were noted to have approximately 1.7- and 2.1-times higher levels of BSP and OC than CA0. Similar trends were noted for the osteogenic profiles of CA10 and CA0. These results showed that the modification of CA was superior in enhancing bone regeneration in a dose-dependent manner. ALP is a common marker used for the prediction of hard tissue regeneration [51]. It has an important role to play in the early stages of mineralization by inducing pulp tissues to form hard tissue matrices. Therefore, it is critically involved in the early stages of regeneration and restoration after dental pulp injuries. In this case, CA20 was noted to be superior in the early stages of regeneration as it has significantly higher levels of ALP after 3 days of culture. On the other hand, BSP is an extracellular matrix of dentin involved in primary mesenchymal cell maintenance and dentin formation. Lastly, OC is a main non-collagenous component synthesized by osteoblast during bone regeneration [52]. Moreover, some studies indicated that aging affected the expression of PTH and PTH1R and thwarted their protein translation that had been shown to regulate and augment the osteogenic differentiation of various hDPSCs [53]. These results showed that CA-modified MTA could enhance in vitro osteogenesis which was hypothesized to be due to increased cellular proliferation and VEGF and Ang-1 expressions as seen above.

### 3.6. In Vivo Bone Regeneration

**Figure 9:**
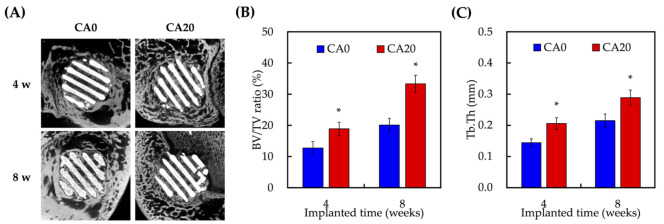
(**A**) Micro-CT image of the bone defects; (**B**) the quantified bone volume/total volume (BV/TV); (**C**) micro-CT quantified trabecular thickness (Tb.Th) analysis at various time points. * indicates a significant difference (*p* < 0.05) when compared to CA0.

To investigate the osteogenic capabilities of our CAMTA scaffolds, 6 mm × 6 mm scaffolds of each group were fabricated using 3D printing and implanted into femur lesions of New Zealand white male rabbits. Micro-CT of the femur and scaffolds were carried out at 4 and 8 weeks post implantation and BV/TV and Tb.Th were evaluated at 4 and 8 weeks post implantation. The relevant results were as shown in Figure 9. After 4 weeks, the micro-CT results showed that there was more bone ingrowth and invasion into the center of the scaffolds in the CA20 group as seen from the heterogeneous tissues amongst the struts of the scaffolds (Figure 9A). As compared to CA0 at 4 weeks, the spaces between the struts were generally hollow with bone growth only at the periphery of the scaffolds. At 8 weeks post implantation, new bones had invaded into the center and struts of the scaffolds. The struts were noted to have fracture lines in the middle due to degradation and new bone regeneration. However, the micro-CT of CA0 only showed increased bone infiltration into the center of the scaffolds. There was obviously more new bone and dense tissue formation in the CA20 group than in the CA0 group. Quantification results including BV/TV (Figure 9B) and Tb.Th (Figure 9C) were recorded at 4 and 8 months post implantation. The BV/TV ratio of the CA20 groups were significantly higher (18.9 ± 2.1% and 33.3 ± 2.7% at 4 and 8 weeks, respectively) as compared with CA0. Similarly, the Tb.Th ratio of the CA20 group was significantly higher (0.21 ± 0.02 mm and 0.29 ± 0.02 mm at 4 and 8 weeks, respectively) than for CA0.

**Figure 10:**
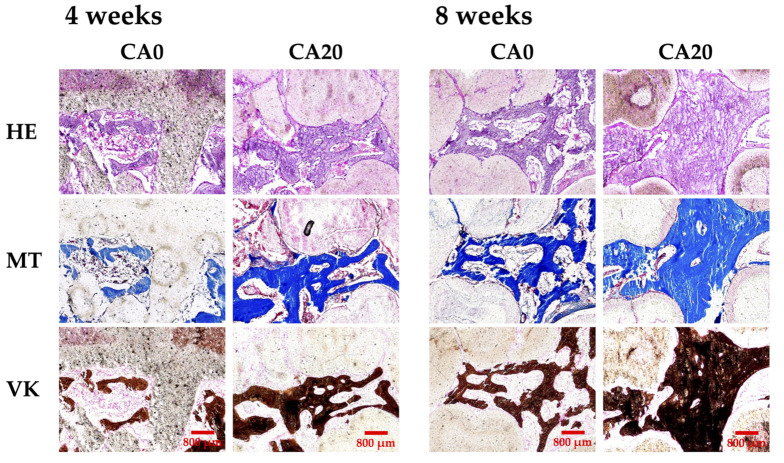
Hematoxylin-eosin (HE), Masson’s trichrome (MT) and von Kossa (VK) staining evaluating new bone regeneration quality of CA0 and CA20 scaffolds in a critical-sized bone defect in vivo at 4 and 8 weeks of implantation. The scale bar is 800 μm.

The scaffolds and the surrounding tissues were harvested at 4 and 8 weeks post implantation and the bone tissue slices were stained with HE, MT and VK staining, as shown in Figure 10. From the HE images, CA20 were found to have the most complete osteogenic-like structures after 8 weeks of implantation as compared to CA0. The VK staining was carried out as a complement to HE staining. As seen, CA20 had darker VK staining as compared to CA0 at all time points, thus indicating higher amounts of calcium deposition or bone formation. The MT images showed formation of complete connective tissues, collagen deposition and neovascularization in the CA20 groups. CA0 had fewer structures and connective tissues than CA20. From the phenomenon observed in the histology, it can be inferred that most of the scaffold around is woven bone after 4 weeks. In addition, the abundant vascular structure, as indicated by the lumen that was found within the woven bone areas in CA20 group. After eight weeks, the VK staining in the CA20 group showed a denser arrangement with fewer lumens, thus we presume that more lamellar bone was formed at this time, which also echoed the previous results. As mentioned, angiogenesis is important for tissue regeneration in providing nutrients and waste removal and new blood vessels bud from existing vascular networks via VEGF signaling. The current result indicated the vascular networks in CA20 were formed around regenerating tissues, thus indicating the need for vascular supply to regenerating tissues [54]. CA20 not only promotes angiogenesis, but also accelerates bone tissue regeneration and mineralization. In fact, numerous studies also had confirmed the antibacterial, anti-inflammatory, anti-carcinogenic and analgesia effects of CA. It was further reported that 30 µM of CA in the surrounding fluid of scaffolds promoted osteogenesis of mesenchymal stem cells by increasing mineralization, ALP and transcription factors [55]. Other reports had also been made stating that CA increases phosphorylated protein kinase B and cyclin D1 production which led to increased stem cell proliferation and osteogenesis [56]. CA has also been used for decades in traditional Chinese medicine, especially for osteoporosis, due to its anti-inflammatory and osteogenesis capability [57]. In the commercial world, biomedical companies and scientists alike are attempting to fabricate an improved version of MTA for clinical application. Currently, MTA is reported to be difficult to manipulate, has prolonged setting duration, maturation phase and discoloration [58]. In our study, it has been proven that modification with CA was able to enhance angiogenic and odontogenic capabilities of MTA. Subsequent studies could involve evaluation of the setting time of our CAMTA and evaluate its potential for creating individualized treatment profiles for each unique individual. In addition, future studies could involve evaluating the discoloration effect of our CAMTA as well as considering other add-on modalities such as growth factors or proteins to evaluate its potential for other applications. With the development of tissue engineering, especially biomaterials, we are now able to modify and combine different biomaterials and compounds in order to increase the regenerative capabilities of the scaffolds. Therefore, the CA-coated MTA scaffolds improved osteogenic characteristics, as compared to pure MTA scaffolds. The current standard treatment for bone defects is using biocompatible biomaterials such as MTA to fill the gap and to provide a platform for regeneration.

## 4. Conclusions

In the present study, the surfaces of the diverse scaffolds were modified by CA coating. Subsequently, we analyze the physicochemical and cell responses regulated by CA coating. In the samples, the CA20 scaffold not only showed good results in mechanical strength but also found apatite precipitate immersed in SBF, which indicated the preferred physical and chemical microenvironment for hDPSCs behaviors. The MTA scaffold coated with CA concentration of 20 mg/mL exhibited promoted cell adhesion, proliferation, and osteogenic differentiation. In addition, VEGF adsorption involved the enhancement of CA coating on osteogenesis differentiation of hDPSCs. Most importantly, MTA scaffold with CA coating showed excellent bone regeneration performance in vivo. Furthermore, the osteogenesis ability of the CA20 scaffold was apparently preferred to that of the CA0 scaffold, which was considered by µCT and histological analysis. These results might be attributed to the enhancement of bone-like apatite formation and VEGF adsorption induced by the CA coated. As a bioinspired polymer, CA can be used as a potently functional bioactive coating for various scaffolds in bone tissue engineering and other biomedical applications.

## Data Availability

Data available in a publicly accessible repository.
